# Extracting causality from spectroscopy

**DOI:** 10.1038/s41598-025-29687-8

**Published:** 2025-12-22

**Authors:** K. Fujita, K. Nakayama, Y. Fujiki, T. Kato, H. Suito, H. Higuchi, T. Sato

**Affiliations:** 1 Computing Laboratory, Fujitsu Research, Fujitsu Limited, Kawasaki, 211- 8588 Japan; 2 Department of Physics, Graduate School of Science, Tohoku University, Sendai, 980-8578 Japan; 3 Frontier Research Institute for Interdisciplinary Sciences (FRIS), Tohoku University, Sendai, 980-8577 Japan; 4 Advanced Institute for Materials Research (WPI-AIMR), Tohoku University, Sendai, 980-8577 Japan; 5 Mathematical Science Center for Co-creative Society (MathCCS), Tohoku University, Sendai, 980-8577 Japan; 6 Mathematical Institute, Graduate School of Science, Tohoku University, Sendai, 980-8578 Japan; 7 Center for Science and Innovation in Spintronics (CSIS), Tohoku University, Sendai, 980-8577 Japan; 8 International Center for Synchrotron Radiation Innovation Smart (SRIS), Tohoku University, Sendai, 980-8577 Japan

**Keywords:** Chemistry, Physics

## Abstract

Causality represents a directed relationship where one state, designated as a cause, directly produces or partially influences another state, an effect. Identifying causality in observations of physical phenomena is a core challenge in science, as it reveals the fundamental laws governing these observations. However, extracting causal relationships from complex data remains difficult. While recent advances in machine learning offer promising avenues, a definitive guiding principle for its application for causal inference has yet to emerge. Here, we propose a protocol to analyze spectroscopy data using DirectLiNGAM, one of statistical causal inferences for learning a Linear Non-Gaussian Acyclic Model. We applied this approach to spatially resolved core-level photoemission spectroscopy measurements of the kagome superconductor CsV₃Sb₅. Our analysis uncovered intriguing causal relationships among Cs surface coverage, core-level intensity/position, and the spectral background. These findings provide an explanation for the polar surface formation in CsV₃Sb₅ and, furthermore, reveal an unexpected causal link in the intensity of spin-orbit satellite peaks. These results highlight the potential of our method to reveal new physical laws that would be difficult to identify using conventional data analysis techniques.

**Supplementary Information:**

The online version contains supplementary material available at 10.1038/s41598-025-29687-8.

## Introduction

**Fig. 1:**
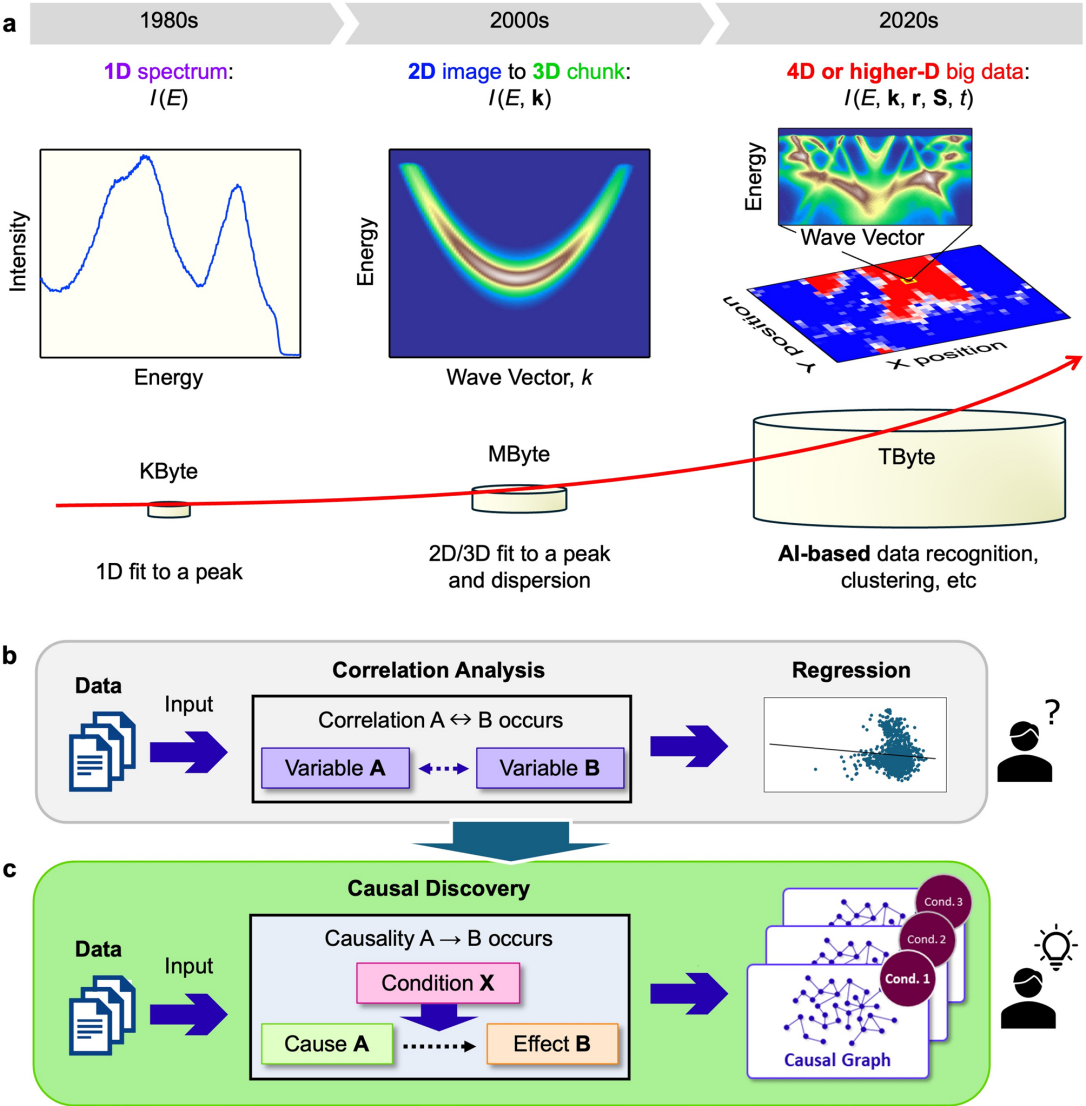
History of photoemission-data quantity and schematics of causal discovery. **(a)** Schematics on the history of data types (top) and data capacity (bottom) in photoemission spectroscopy. In the 1980s, photoemission data were mainly recorded as a 1D spectrum, *I*(*E*). In the 2000s, due to the development of advanced electron analyzers that enable the simultaneous angular collection of photoelectrons, photoemission data were obtained as a 2D image, *I*(*E*, *k*_1_). Further, acquiring a series of 2D images enabled the construction of 3D chunk data *I*(*E*, *k*_1_, *k*_2_). By the 2020s, due to an advancement of highly brilliant micro/nano-spot light sources together with the development of state-of-the-art spin detectors, photoemission data contain multi-dimensional (more than 4D) elements, including lateral position (*x*, *y*) on the sample surface and spin component (*S*_*x*_, *S*_*y*_, *S*_*z*_). Consequently, data capacity increased from Kbytes (in the 1980s) to Gbytes/Tbytes (in the 2020s). **(b)** Schematic of a conventional data analysis flow. Researchers or artificial intelligence (AI)-based approaches extract correlations between input variables, from which researchers try to estimate causality using regression models or by relying on their intuition and experience. **(c)** Schematic of AI-based causal discovery from the data. AI-based methods identify causal relationships, i.e., cause variables and their corresponding effect variables with/without predefined conditions, and generate causal graphs without expert intuition or experience. Researchers can notice underlying scientific laws from these causal graphs.

Recent groundbreaking discoveries in science have been fueled largely by advancements in experimental techniques. These advancements have significantly enhanced both the quantity and quality of experimental data, resulting in an era of unprecedented data richness. For example, photoemission spectroscopy, a powerful method for probing the electronic structure of solids^1,2^, measured photoelectron intensity (*I*) only as a function of energy (*E*) in the 1980s. By the 2000s, however, it gained the ability to resolve electron momentum (**k**), enabling the visualization of band dispersion and the Fermi surface, followed by the more recent development of spatial (**r**), spin (**S**), and/or time (*t*) resolved measurements (Fig. 1a). These innovations have expanded data acquisition from one-dimensional (1D) spectra to two-dimensional (2D) images, three-dimensional (3D) datasets, and ultimately higher-dimensional big data, accompanied by a substantial increase in data volume. The surging dimensionality and volume of experimental data have driven progress in data analysis techniques, evolving from 1D peak fitting to multi-dimensional fitting and, more recently, to machine-learning-based methods such as data recognition and clustering^3–17^. Even when dealing with large datasets, these modern approaches enable in-depth analysis of correlations among various physical quantities with minimal human intervention (Fig. 1b). Nevertheless, identifying causal relationships—crucial for elucidating the mechanisms behind physical properties and uncovering fundamental scientific laws—still heavily depends on expert knowledge and experience.

A prime example where simple correlation analysis proves insufficient is the decades-long debate about the origin of the “pseudogap” in copper-oxide high-temperature superconductors. For many years, different angle-resolved photoemission spectroscopy (ARPES) groups consistently reported a robust correlation: a spectral gap (pseudogap) that opens at a temperature T* above the superconducting transition temperature (*T*_c_) exhibits a similar anisotropy in momentum space to the well-known *d*-wave superconducting gap below *T*_c_^18,19^. However, this correlation was consistent with two fundamentally different and mutually exclusive causal models. One model proposed that the pseudogap is a precursor to superconductivity. In this causal scenario, the similar momentum dependence is interpreted as evidence for preformed Cooper pairs that exist above *T*_c_. The other model argued that the pseudogap is a manifestation of a competing order, such as a charge order, which opposes superconductivity. In this causal picture, the pseudogap actively suppresses superconductivity by removing the density of states available for Cooper pairing. Crucially, the fundamental ARPES observation—namely, the strong correlation in the momentum-space anisotropy of the two gaps—was insufficient to distinguish between these competing scenarios. This ambiguity in causality was the fundamental reason why the debate persisted for over two decades. This historical example highlights that extracting causality from modern spectroscopy data based solely on human intuition is fraught with ambiguity and therefore requires the development of systematic causal discovery frameworks.

Developing methods to elucidate causal relationships between physical quantities is a key objective in various fields. Conducting controlled experiments can be effective, but practical limitations, such as cost constraints and difficulties in preparing well-controlled samples, make them infeasible. Consequently, several methods for causal inference from observational data have been explored. Structural equation models (SEMs)^20^ are widely used in causal analysis, with linear acyclic SEMs being particularly useful for analyzing continuous variables. One such model is the Linear non-Gaussian acyclic model (LiNGAM)^21^, which assumes non-Gaussian noise distributions. LiNGAM can generate a directed acyclic graph (DAG; Fig. 1c) that represents causal orderings and connection strengths between variables, without requiring prior knowledge of the DAG structure.

In this work, we propose a method to extract causality from ARPES measurement data using DirectLiNGAM, a powerful algorithm for direct estimation of LiNGAM^22^. Our method also includes a technique for inferring regions with characteristic causal relations based on efficient enumeration of conditions in the measurement data^23^. We demonstrate the effectiveness of this method by applying it to spatially resolved ARPES data for two different materials, a kagome superconductor CsV_3_Sb_5_^24,25^ and a topological insulator [(PbSe)_5_][(Bi_2_Se_3_)_3_]_4_^26^. Our analysis reveals several important causal relationships, providing insights into the mechanisms of physical properties of these materials.

Our approach is distinct from conventional spectroscopic calculations. Such spectral calculations are a physics-driven approach, and begin with a pre-defined physical model, e.g., using density functional theory incorporating many-body effects, to theoretically reproduce spectra and thereby elucidate the microscopic origins of observed phenomena. However, constructing such a model for a highly complex system like that in this study is a challenging task. In contrast, our LiNGAM-based method is data-driven, and infers causal relationships directly from observational data without requiring a prior construction of a physical model. This makes it a powerful tool for generating hypotheses and providing new perspectives in complex systems where an appropriate physical model has yet to be established. We expect that these two approaches can be complemental. For example, causal relationships discovered by our method could provide constraints and guidance for the development of accurate physical models, and these models could then be used in spectroscopic calculations to verify the underlying mechanisms.

## Results and discussion

### The entire flow of causal discovery

**Fig. 2:**
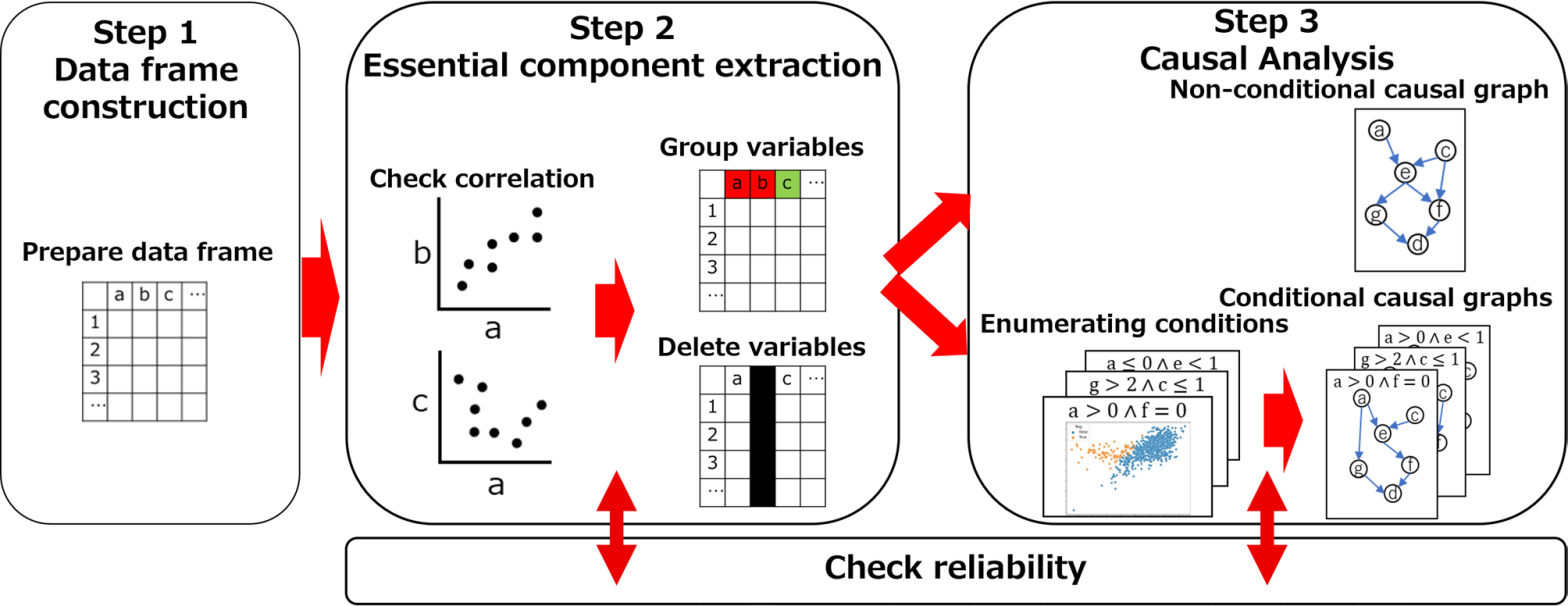
The flow of causal discovery. The proposed scheme in this study consists of three main steps: (Step 1) The input data are prepared. (Step 2) The correlations in the input data are analyzed. When highly correlated variable pairs are identified, one variable is removed to reduce complexity in the subsequent causal discovery process. (Step 3) Causality is inferred, and causal graphs are generated. In Step 2 and 3, the reliability of variable removal and causal relationships are assessed.

The proposed causal discovery scheme, outlined in Fig. 2, consists of three main steps.

Step 1, Data frame construction, prepares the input data, where rows represent data samples and columns correspond to variables, which become nodes in causal graphs generated in the following steps. Step 2, Essential component extraction, selects essential variables from the prepared data frame by removing unnecessary ones. In general, a larger number of nodes in a generated causal graph makes the understanding of its meaning more difficult. To enhance the clarity of a causal graph, this step analyzes correlations between variables, identifies highly correlated pairs, and removes one variable in each pair if its removal does not significantly change the causal graph. Step 3, Causal analysis, infers causal relationships from the data consisting of essential variables, generating causal graphs. In this step, a non-conditional causal graph using all the samples and conditional causal graphs for selected characteristic conditions are obtained. A causal graph is a weighted directed graph, where edges and their directions represent causal relationships and the weights of the edges indicate the strength of causal effects. In addition, edge reliability is assessed by the bootstrap method^27^.

## Data frame construction from ARPES results of a kagome superconductor

**Fig. 3:**
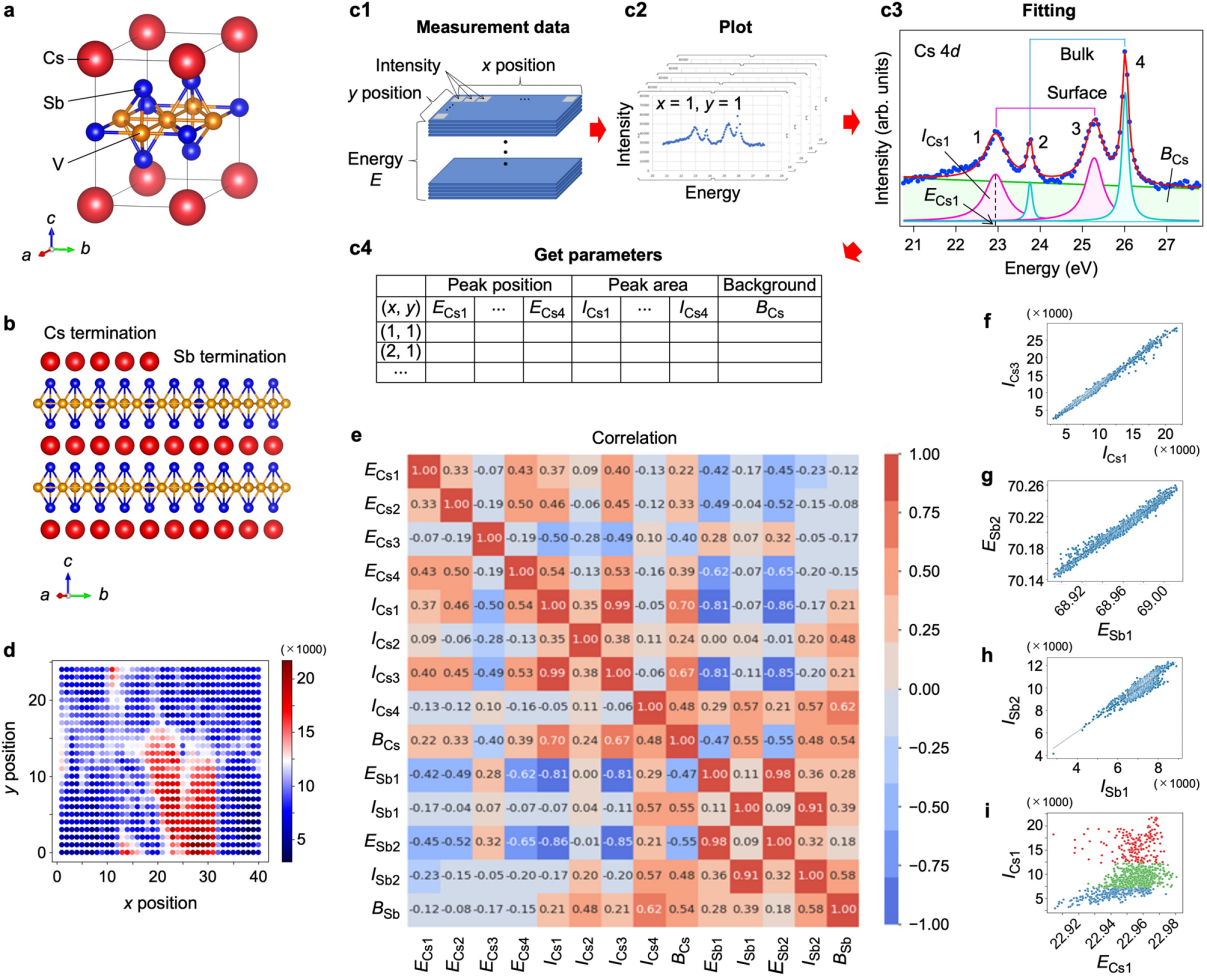
Data frame construction and essential variable extraction from the photoemission data. **(a)** Crystal structure of CsV_3_Sb_5_. **(b)** Side view of the crystal structure with Cs- and Sb-terminated surfaces. **(c)** Schematics of the procedure to construct the input data frame in this study. In panel **c3**, a representative photoemission spectrum (dots) for the Cs 4*d* core levels is displayed, with the result of numerical fittings (solid curves) with four Lorentzian peaks and a linear background. Peak 1 and 3 represent spin-orbit partners for surface-derived peaks, while peak 2 and 4 represent those for bulk-derived peaks. **(d)** Spatial mapping of total spectral weight of peak 1 for Cs core levels, which highlights the existence of Cs-rich (red) and Sb-rich (blue) domains. **(e)** Correlation matrix for all the variables, i.e. spectral weight and peak energy for Cs core levels (*I*_Cs1_-*I*_Cs4_ and *E*_Cs1_-*E*_Cs4_, respectively) and those for Sb core levels (*I*_Sb1_-*I*_Sb2_ and *E*_Sb1_-*E*_Sb2_, respectively), together with their background intensity (*B*_Cs_ and *B*_Sb_). **(f**-**i)** Scatter plots of (*I*_Cs1_, *I*_Cs3_), (*E*_Sb1_, *E*_Sb2_), (*I*_Sb1_, *I*_Sb2_), and (*E*_Cs1_, *I*_Cs1_) pairs, respectively. In (**i**), data points corresponding to Cs-rich, intermediate, and Sb-rich regions are indicated by red, green, and blue dots, respectively (see Fig. 5b).

We applied our causal discovery method to the spatially resolved core-level photoemission spectra of CsV_3_Sb_5_. As shown in Fig. 3a, CsV_3_Sb_5_ consists of alternating Cs layer and V_3_Sb_5_ layers, with the latter containing the V-based kagome lattice responsible for exotic physical properties such as superconductivity and charge-density wave^24,25,28–31^. Due to strong V-Sb bonding, the crystal surface probed by photoemission is terminated by either Cs or Sb atoms (Fig. 3b; note that Fig. 3b illustrates two extreme cases, but in reality, the amount of residual Cs atoms on the surface exhibits spatial variation, as discussed later)^32–35^. The core-level spectra of CsV_3_Sb_5_ serve as a good platform to apply our method because the well-defined peaks allow precise extraction of peak parameters (such as peak position, width, and weight), as shown later. Also, the data are known to show marked spatial variations^34,35^ which could be useful to explore the relationship between non-conditional and conditional causal relationships.

Figure 3c illustrates details of the data processing in Step 1 (Fig. 2). First, we prepared two datasets: Cs and Sb core levels, each of which is a matrix of photoemission intensity recorded as a function of energy (*E*) and spatial coordinates (*x* and *y*), with dimensions of 124 (along *E*) × 40 (*x*) × 25 (*y*) cells (Fig. 3c1). Second, we plotted spectral intensity as a function of energy, *I*(*E*), at each pair of (*x*, *y*) position (Fig. 3c2). A representative Cs 4*d* core-level spectrum (dotted curve in Fig. 3c3) shows four peaks: spin-orbit satellite peaks (called here peak 2 and 4, respectively) from bulk Cs atoms embedded between the V_3_Sb_5_ layers in Fig. 3b and those from surface counterparts (peak 1 and peak 3) (see Supplementary note 1 for the verification of the number of peaks). Next, we carried out numerical fittings with four Lorentzian peaks with a linear background (Fig. 3c3), described by:

$$\:\sum\:_{i=1}^{n}\left(\frac{{c}_{i}}{{\left(E-{a}_{i}\right)}^{2}+{b}_{i}^{2}}\right)+d\cdot \:E+e$$

where $\:n$ is the number of peaks, which is four for Cs core-level spectra. Regarding physical constraints, we imposed the condition *d*⋅*E* + *e* ≥ 0 to ensure the linear background term remains non-negative. Apart from this, we did not impose additional constraints on parameters such as a fixed weight ratio between spin-orbit partners. This is because, as we discuss later in detail, the weight ratio of peaks is not constant across the sample surface. The numerical fitting (solid curve) well traces the experimental core-level spectrum (dots), indicating that our numerical fittings are of satisfactory. As a consequence, we obtained initial input variables (Fig. 3c4): peak position ($\:{a}_{i}$) of Cs core levels, called here *E*_Cs1_-*E*_Cs4_, for peaks 1–4, respectively; spectral weight ($\:{c}_{i}/{b}_{\text{i}}$) labeled as *I*_Cs1_-*I*_Cs4_ for peaks 1–4, respectively; total background intensity (in the energy range observed) labelled as *B*_Cs_. We have used the same labelling scheme to Sb 4*d* core levels consisting of two peaks (*n* = 2)^34^, and extracted parameters *E*_Sb1_, *E*_Sb2_, *I*_Sb1_, *I*_Sb2_, and *B*_Sb_. In total, we have obtained 14 variables, 9 for Cs, and 5 for Sb core levels.

These variables represent genuine physical quantities. For instance, *E*_Cs1_-*E*_Cs4_, *E*_Sb1_, and *E*_Sb2_ correspond to the electron’s binding energies, and the energy splitting between spin-orbit partners, (*E*_Cs1_, *E*_Cs3_), (*E*_Cs2_, *E*_Cs4_), and (*E*_Sb1_, *E*_Sb2_), is a well-established physical effect. Regarding the spectral weight, it can be influenced by the photo-ionization cross-section (matrix element). However, because the key experimental conditions (photon energy, polarization, photoelectron emission angle, crystal orientation, etc.) were fixed during the measurement, the matrix element should not vary across the different positions. Therefore, relative changes in the peak weight can be considered proportional to the local concentration (or coverage) of the corresponding atoms (e.g., Cs). It should be also noted that we initially included peak width and peak intensity as separate variables. However, the resulting causal graph became difficult to interpret because of the large number of nodes and edges. To simplify the model and enhance interpretability, we chose to combine these parameters into a single variable, the spectral weight. This reduction in parameters proved to be a critical step, leading to a significant improvement in our ability to interpret the results.

As a verification of the correctness of our variable extraction, we plot in Fig. 3d a heatmap of *I*_Cs1_ in real-space coordinates (*x*, *y*). Since *I*_Cs1_ is the spectral weight of the surface-derived Cs core-level peak as described above, it should reflect the concentration of remaining Cs atoms on the local surface area. The clear spatial variations observed in Fig. 3d indicate spatial inhomogeneity in the surface Cs concentration. Specifically, high-intensity (red) and low-intensity (blue) regions correspond to Cs-rich and Sb-rich domains, respectively. The spatial distribution of these domains is consistent with that obtained from raw spectral intensity map^34^, confirming the validity of our variable extraction. We also confirmed the validity of our variable extraction through a comparison with the result obtained using a different fitting model, as discussed later.

## Essential component extraction

In Step 2, we first calculated the Pearson correlation coefficient (*r*) for all 14 input variables (Fig. 3e). Several variable pairs exhibit a high *r* value over 0.90. The highest correlation (*r* = 0.99) is observed for the (*I*_Cs1_, *I*_Cs3_) pair, as also evident in the corresponding scatter plot in Fig. 3f. This strong correlation is physically reasonable because *I*_Cs1_ and *I*_Cs3_ represent the spectral weights of spin-orbit partners of surface-originated Cs 4*d* states, which should be proportional with each other. Similarly, the second highest correlation (*r* = 0.98) is found for the (*E*_Sb1_, *E*_Sb2_) pair (its scatter plot is displayed in Fig. 3g), reflecting the expected correspondence between the energy shifts of spin-orbit partners for the Sb 4*d* orbital. The strong correlation between the Sb core levels is also reflected as a high *r* (0.91) of the (*I*_Sb1_, *I*_Sb2_) pair (see also the scatter plot in Fig. 3h). These highly correlated three pairs are distinct from other variable pairs that do not clearly follow a linear relationship, as highlighted by the scatter plot for the (*E*_Cs1_, *I*_Cs1_) pair in Fig. 3i (see also Supplementary note 2 for other pairs).

Next, we grouped the strongly correlated variables, (*I*_Cs1_, *I*_Cs3_), (*E*_Sb1_, *E*_Sb2_), and (*I*_Sb1_, *I*_Sb2_), by setting a threshold of *r* ≥ 0.90. To simplify the interpretation of causal graphs, one variable was removed from each group, i.e. *I*_Cs1_, *E*_Sb1_, and *I*_Sb1_. This reduced the number of variables from 14 to 11. It is noted that, with this threshold (*r* ≥ 0.90), removing the selected variables, or their alternatives *I*_Cs3_, *E*_Sb2_, and *I*_Sb2_, does not essentially influence the resulting causal graph. However, removing a variable from a pair with *r* < 0.90 can influence, indicating that such groupings are inappropriate, as detailed in Supplementary note 3.

## Causal analysis

**Fig. 4:**
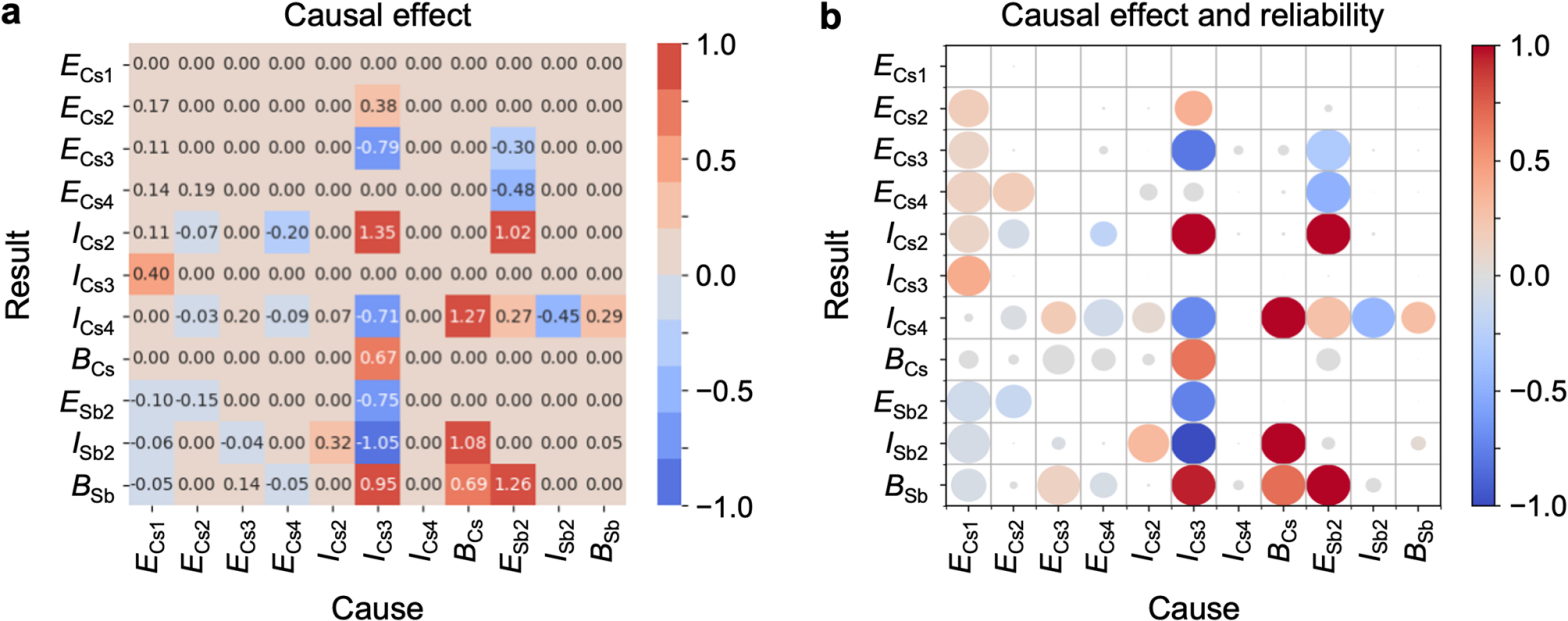
Causal effects obtained from the photoemission data. **(a)** Adjacency matrix between “result” and “cause” variables after removing *I*_Cs1_, *E*_Sb1_, *I*_Sb1_. The values in the matrix represent causal effects. **(b)** Same as **(a)**, but the magnitude of causal effects is highlighted by the gradual color shading of circles. The size of circles corresponds to the probability of the corresponding causal relationship in bootstrap resampling (larger size indicates higher probability).

Proceeding to Step 3, we analyzed causal relationships among the 11 essential variables and generated an adjacency matrix in Fig. 4a. In this matrix, each row represents a “result” variable, and each column corresponds to a “cause” variable. The values in the matrix, indicating the causal effect or the connection strength (called *b*), quantify the change in a “result” variable per unit change in a “cause” variable. For comparison, all matrix values were standardized: that is, for each variable, an originally given value *v* was normalized by $\:\left(v-\mu\:\right)/\sigma\:$, where $\:\mu\:$ and $\:\sigma\:$ are the average and standard deviation of all the values for the variable. Figure 4b visualizes these *b* values using gradual color-coded circles. The circle radii indicate the probability of each causal relationship, evaluated by 100 times causal discovery using the bootstrap method (larger circles denote higher probability). Overall, when the absolute *b* value is high, the probability is also high, validating the data sampling for causal inference. Notably, *I*_Cs3_ and *E*_Sb2_ exhibit large influences on many other variables, acting as the primary and secondary key “cause” parameters, respectively. On the other hand, 79 out of 121 (11 × 11) of *b* values, corresponding to 65% of the entry, were zero. *I*_Cs4_ consistently show no causal influence as a “cause” variable, as all *b* values in its columns are zero. Low *b* values are also observed for the *I*_Cs2_ and *B*_Sb_ columns. Conversely, *I*_Cs4_, *I*_Cs2_, and *B*_Sb_ exhibit relatively high *b* values as “result” variables. These results indicate that the spectral weight of the bulk-originated Cs core-level peaks and the background intensity of the Sb core levels are more likely to be “results” rather than “causes”.

**Fig. 5:**
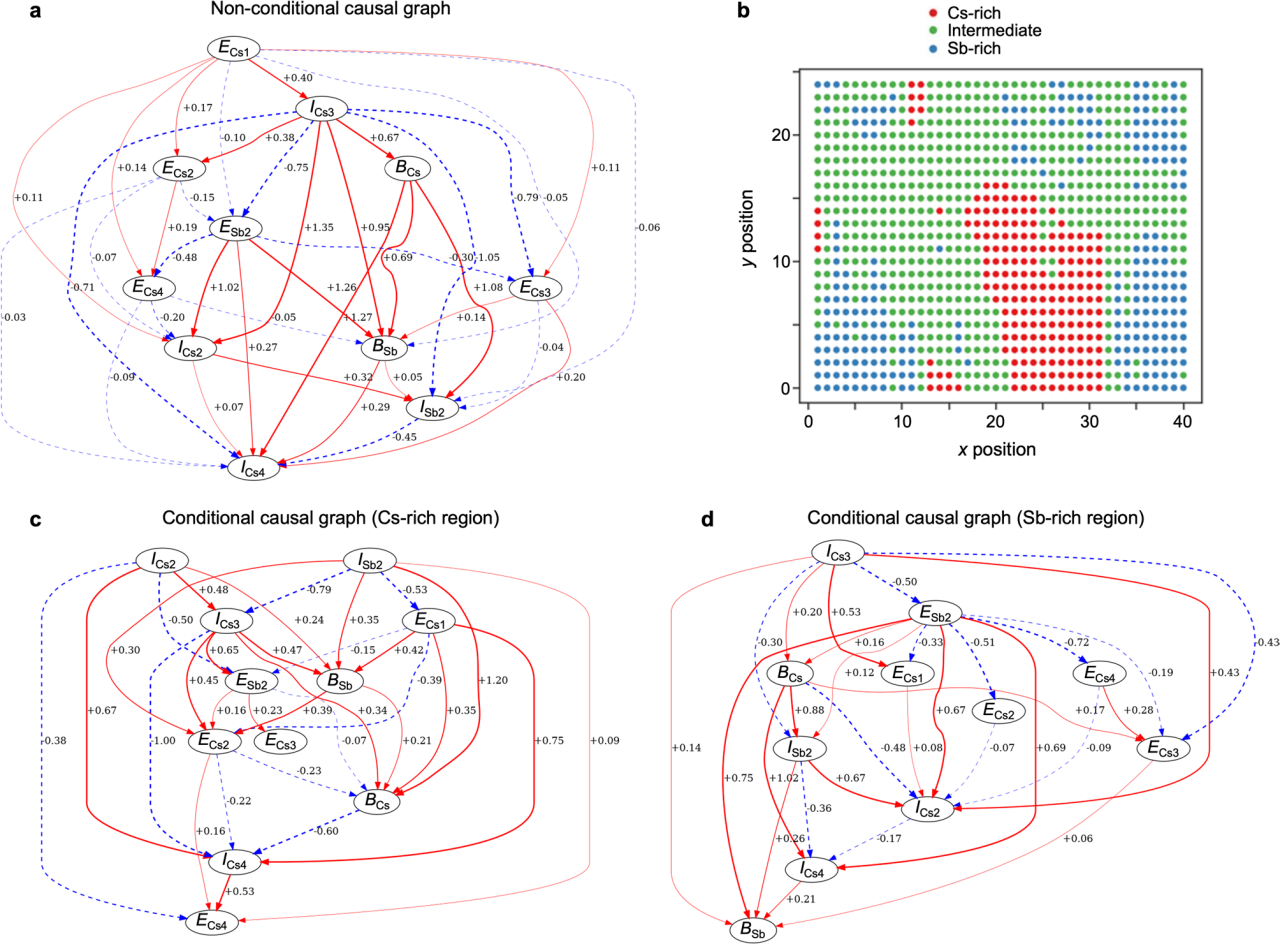
Comparison of non-conditional and conditional causal graphs for a kagome superconductor CsV_3_Sb_5_. (**a**) Causal graph obtained from data in full (*x*, *y*) region. Thicker edges indicate stronger causal relationships between the connected two variables, and red and blue edges represent positive and negative causal effects, respectively. (**b**) Distribution of Cs- and Sb-rich domains (red and blue dots, respectively) on the sample surface, based on clustering of the scattering plot for *E*_Cs1_ vs. *I*_Cs1_ in Fig. 3i. (**c**,**d**) Causal graphs obtained from data at the (*x*, *y*) positions indicated by red and blue dots, respectively, in (**b**).

To better visualize the causal relationships in the adjacency matrix (Fig. 4a), we generated a non-conditional causal graph (Fig. 5a). A causal graph in a LiNGAM^19^ is a directed acyclic graph, in which each variable in the input data frame appears as a node, and directed edges (arrows) depict inferred causal relationships. The source node of an edge corresponds to the “cause” variable, while the sink node represents the “result” variable. The edge weight, equivalent to *b* in Fig. 4a, is reflected in edge thickness, and the edge color (red or blue) indicates the sign of *b*.

In addition to the non-conditional causal relationships derived from the entire surface area (Fig. 5a), we have also investigated conditional causal relationships based on Cs surface coverage. Given the significant spatial variation in Cs concentration at the surface (Fig. 3d), we have categorized the input data into three regions based on the *I*_Cs1_ value, i.e. Cs-rich (red dots in Fig. 5b), Sb-rich (blue dots), and intermediate region (green dots). The resulting conditional causal graphs for the Cs-rich and Sb-rich domains are shown in Fig. 5c, d, respectively. It should be noted that the causal graphs in Fig. 5c, d are displayed with an auto-generated layout, as is typically produced by the LiNGAM algorithm (see Supplementary note 4 for graphs in which each node has been manually arranged to maintain consistent positions).

One can observe common features in the non-conditional and conditional causal graphs (Fig. 5a, c, d). For example, focusing on direct effects of *I*_Cs3_, which lies near the top of the graphs and emits multiple edges toward other variables as previously noted (Fig. 4a), we find that a prominent edge from *I*_Cs3_ to *E*_Sb2_ consistently appears in all graphs. This indicates variations in *I*_Cs3_ (and equivalently *I*_Cs1_), reflecting surface Cs concentration (Fig. 3d), directly influence the shift of the Sb core-level energy (*E*_Sb2_). This causality is significant, as it supports a key property of CsV_3_Sb_5_, namely, that increased surface Cs coverage leads to electron donation into the V_3_Sb_5_ layer, reflecting the polar nature of its surface^34,35^. Furthermore, unlike previous studies that suggested the similar causality based on researchers’ knowledge^34,35^, the present scheme reached the same conclusion without such expert knowledge. This successful and independent reproduction of the known relationship serves as a crucial benchmark for validating the ability of LiNGAM to extract physically meaningful causality (see Supplementary notes 5,6 for analyses of the robustness of the obtained causalities; see Supplementary note 7 for a comparison between LiNGAM and other algorithms).

Another noteworthy observation in causal graphs is associated with the background intensity. An edge commonly links *I*_Cs3_ to *B*_Sb_ in all causal graphs. This suggests that the spectral weight of surface-derived Cs core level influences the background intensity of the Sb core levels. Given the fact that energies of the Cs 4*d* core levels (23–26 eV) are much lower than those of Sb 4*d* (69–70 eV), a conventional Shirley-type background associated with secondary electrons^36^ is unlikely to explain this relationship. Instead, we propose that photoelectron scattering from surface Cs atoms may increase the background, besides the conventional Shirley-type background. This hypothesis is supported by the weakened causal effect (thinner edge) observed in the Sb-rich domain (Fig. 5d) compared to the Cs-rich domain (Fig. 5c), consistent with reduced electron scattering due to sparse surface Cs atoms. We emphasize that such insights into the causal relationships between core-level intensity and background are difficult to obtain without data analysis based on causal inference, highlighting the benefit of the unique integration of spectroscopy and causal discovery techniques in this study.

Besides these interpretable results, an unexpected causal relationship between *I*_Cs2_ and *I*_Cs4_ is observed upon careful inspection of the causal graphs. Given the strong correlation between the spin-orbit partners for surface-originated Cs core-level peaks (*I*_Cs1_ and *I*_Cs3_; Fig. 3e, f), one would expect a similar correlation between their bulk counterparts, *I*_Cs2_ and *I*_Cs4_. However, *I*_Cs2_ and *I*_Cs4_ exhibit a surprisingly low correlation (*r* = 0.11; see Fig. 3e). Intriguingly, *I*_Cs2_ shows a positive causal effect (red arrow) on *I*_Cs4_ in the non-conditional case (Fig. 5a) and the Cs-rich case (Fig. 5c), but a negative effect in the Sb-rich case (Fig. 5d). This indicates a significant variation in the *I*_Cs2_/*I*_Cs4_ ratio depending on sample position (*x*, *y*) and domain type (Cs-rich or Sb-rich). This finding contradicts the textbook characteristics of the spin-orbit satellite peaks, where the weight ratio, which is in principle quantum-mechanically determined by electron occupation, should remain constant (the violation of this relationship is confirmed by additional data analyses in Supplementary note 8). While mechanisms such as final-state effects, photo-ionization cross-sections, Auger processes, and resonance photo-excitations may alter this ratio^37^, they cannot explain the observed variation because these effects must be insensitive to the probed surface area. The origin of this unexpected phenomenon is beyond our current understanding, and we leave it as an open question. Nonetheless, this highlights the potential of combining causal discovery with spectroscopy to discover novel physics law that can hardly be obtained by conventional human-based analysis. It is noted that the unusual *I*_Cs2_/*I*_Cs4_ ratio discussed above could be also revealed by a conventional correlation analysis. However, causal inference uncovers that *I*_Cs2_ is the cause of *I*_Cs4_. This is the crucial distinction between the correlation and causal inference analyses, and provides a guide for future investigations. For example, it suggests that any physical model seeking to explain the Cs core-level spectrum should treat the mechanism responsible for the peak 2 as the primary driver. In this way, causal inference acts as a useful tool to narrow down hypotheses to construct a detailed physical model. Furthermore, this method is applicable to other systems, as shown in Supplementary note 9 for a topological insulator [(PbSe)_5_][(Bi_2_Se_3_)_3_]_4_^26,38^, and can be extended to datasets obtained by other spectroscopy techniques.

## Methods

### LiNGAM

 LiNGAM is a type of structural equation models (SEMs) and represents a data generating process with a directed acyclic graph (DAG). For a p-dimensional vector $\:\varvec{x}\:=\:{\left({x}_{1},\:.\:.\:.\:,\:{x}_{p}\right)\:}^{T}\:\in\:\:{\mathbb{R}}^{p}$, we consider a weighted adjacency matrix of a DAG with $\:p$ nodes $\:\varvec{B}\:=\:{\left({b}_{ij}\right)}_{p\times\:p}\:\in\:\:{\mathbb{R}}^{p\times\:p}$. Each element $\:{b}_{ij}$ represents the direct causal effect from variable $\:{x}_{j}$ to another $\:{x}_{i}$ in the DAG. Without loss of generality, each observed variable $\:{x}_{i}$ is assumed to have zero mean. Then, the following equation expresses LiNGAM:

$\:\varvec{x}\:=\:\varvec{B}\varvec{x}\:+\:\varvec{e}$, where $\:\varvec{e}\:=\:{\left({e}_{1},\:.\:.\:.\:,\:{e}_{p}\right)}^{T}\:\in\:\:{\mathbb{R}}^{p}$ is a random noise vector. We assume that each $\:{e}_{i}$ has a non-Gaussian distribution with zero mean and a non-zero variance. Furthermore, all $\:{e}_{i}$ are assumed to be independent of each other so that there are no latent confounding variables. DirectLiNGAM^20^ is an algorithm for estimating a causal ordering and the connection strengths in the LiNGAM.

#### Lemma 1

^20^: *Assume that the input data* **x** *strictly follows the LiNGAM. Denote by* $\:{r}_{i}^{\left(j\right)}$ *the residuals when* $\:{x}_{i}$ *are regressed on* $\:{x}_{j}:\:{r}_{i}^{\left(j\right)}\:=\:{x}_{i}\:-\frac{cov\left({x}_{i},{x}_{j}\right)}{var\left({x}_{j}\right)}{x}_{j}\:(i\ne\:j)$. *Then a variable* $\:{x}_{j}$ *is exogenous if and only if* $\:{x}_{j}$ *is independent of its residuals* $\:{r}_{i}^{\left(j\right)}$ *for all* $\:i\ne\:j$.

#### Lemma 2

^20^: *Assume that the input data* ***x*** *strictly follows the LiNGAM. Furthermore, assume that a variable* $\:{x}_{j}$ *is exogenous. Denote by* $\:{r}^{\left(j\right)}\:$*a vector collecting the residuals* $\:{r}_{i}^{\left(j\right)}$ *by* $\:{x}_{j}$ *for all* $\:{x}_{i}\:(i\ne\:j)$. *Then LiNGAM is preserved on* $\:{\varvec{r}}^{\left(j\right)}:\:{\varvec{r}}^{\left(j\right)}\:=\:{\varvec{B}}^{\left(j\right)}\:{\varvec{r}}^{\left(j\right)}\:+{\varvec{e}}^{\left(j\right)}$, *where* $\:{\varvec{B}}^{\left(j\right)}$ *is a matrix that can be permuted to be strictly lower-triangular by a simultaneous row and column permutation, and elements of* $\:{\varvec{e}}^{\left(j\right)}$ *are non-Gaussian and mutually independent*.

#### Corollary 1

^20,39^: *Assume that the input data* ***x*** *strictly follows LiNGAM. Furthermore, assume that variable* $\:{x}_{j}$ *is exogenous. Then, for any causal order* $\:\mathcal{C}\mathcal{O}$ *of* $\:\varvec{x}$, *there is a causal order* $\:\mathcal{C}{\mathcal{O}}_{j}$ *of* $\:{\varvec{r}}^{\left(j\right)}$*such that* $\:\mathcal{C}\mathcal{O}\left(k\right)<\mathcal{C}\mathcal{O}\left(l\right)\iff\:\mathcal{C}{\mathcal{O}}_{j}\left(k\right)<\mathcal{C}{\mathcal{O}}_{j}\left(l\right)$ *holds for any* $\:k\ne\:j$ *and* $\:l\ne\:j$, *i.e*., $\:{\varvec{r}}^{\left(j\right)}$ *preserves the possible causal orders of* ***x***.

By Lemma 2 and Corollary 1, we observe that a causal order can be estimated by recursively identifying an exogenous variable from the residuals. That is, if the algorithm selects $\:{x}_{j}$ as an exogenous variable in the current step, then the algorithm replaces ***x*** by $\:{\varvec{r}}^{\left(j\right)}$ before proceeding to the next step and removes $\:{x}_{j}$. This operation is repeated until all variables are selected. Consequently, $\:\mathcal{C}\mathcal{O}\left(i\right)$ represents the number of steps before $\:{x}_{i}$ is selected.

To apply Lemma 1, we need to use a measure of independence. A common independence measure between two variables $\:{y}_{1}$ and $\:{y}_{2}$ is their mutual information $\:I\left(x,y\right)$^40^. Let $\:K\:\subseteq\:\:\left[p\right]$ be the subset of indices whose corresponding variables are unordered. For any $\:i,\:j\:\in\:\:K,\:\:{M}_{ij}\::=\:I\left({x}_{j},\:{r}_{i}^{\left(j\right)}\right)\:-I\left({x}_{i},\:{r}_{j}^{\left(i\right)}\right)$ indicates the precedence between $\:{x}_{i}$ and $\:{x}_{j}$ as follows: $\:{x}_{i}$ precedes $\:{x}_{j}$ if $\:{M}_{ij}\:>\:0$, the precedence between $\:{x}_{i}$ and $\:{x}_{j}$ is arbitrary if $\:{M}_{ij}\:=\:0$, and $\:{x}_{j}$ precedes $\:{x}_{i}$ if $\:{M}_{ij}\:<\:0$. Let $\:{m}_{i}\::={{\Sigma\:}}_{j\in\:K,i\ne\:j}\:\text{m}\text{i}\text{n}{\left(0,{M}_{ij}\right)\:}^{2}$. Then, because exogenous variables should precede more variables, the algorithm selects $\:{x}_{{j}^{*}}$ as an exogenous variable for $\:{j}^{*}\:\in\:\:\text{a}\text{r}\text{g}\:{\text{m}\text{i}\text{n}}_{j\:\in\:K}{m}_{j}$.

After estimating a causal order $\:k$, the algorithm estimates an adjacency matrix ***1*** as follows: $\:{b}_{ij}$ becomes a coefficient of a sparse regression such that the precedent variables $\:\left\{{x}_{j}\:\right|k\left(j\right)\:<\:k\left(i\right)\}$ regress $\:{x}_{i}$. Adaptive LASSO^41^ is a recommended sparse regression method for this purpose.

### Data frame construction

 We used the SciPy library optimize.least_squares^42^ to fit the photoemission data^34^ with Lorentzian functions of the form

$$\:\sum\:_{i=1}^{n}\left(\frac{{c}_{i}}{{\left(E-{a}_{i}\right)}^{2}+{b}_{i}^{2}}\right)+d\cdot \:E+e$$.

To prevent the linear background term $\:d\cdot \:E+e$ from taking unphysical negative values, we imposed the constraint $\:d\cdot \:E+e\ge\:0$. We performed the fitting using this formula and obtained the parameters $\:{a}_{i},\:{b}_{i},\:{c}_{i}\:(i=\text{1,2},\dots\:,n)$, $\:d$, and $\:e$. These parameters were then used to construct a data frame of essential components, as described in the Essential component extraction section of the main text.

### Causal discovery

We chose the DirectLiNGAM algorithm for our causal analysis because its underlying assumptions, such as linearity, non-Gaussianity, acyclicity, and the absence of unobserved confounders, are justified for our spatially-resolved core-level photoemission data. Regarding the absence of confounding factors, it is generally recognized that confounding effects leading to the simultaneous energy shift and spectral-weight changes in core-level peaks are not present. Furthermore, cyclic relationships are not observed. With respect to linearity, as illustrated by the scatter plots for each variable pair in Fig. S2, while some cases exhibit high variability or a few points deviating from a strict linear trend, no pairs demonstrate strong nonlinearity. To evaluate robustness to Gaussian noise, we assessed the reliability of the inferred causal relations using their probabilities of appearance across bootstrap samples.

DirectLiNGAM object was created with the Python LiNGAM package^43^, and the fit method was called using the data frame to generate a non-conditional causal graph. To draw conditional causal graphs, we extracted one or more characteristic conditions from the same data frame. For each condition, a data frame satisfying the condition was created, and a corresponding conditional causal graph was generated by calling the fit method of DirectLiNGAM. Specifically, as shown in Fig. 3d, the surface area was divided into Cs-rich, Sb-rich, and intermediate domains based on the surface Cs concentration. Accordingly, we narrowed down the extraction to the conditions classified by *I*_Cs1_. In Fig. 3i, where the horizontal and vertical axes are *E*_Cs1_ and *I*_Cs1_, respectively, the data is divided into two parts: one with a high correlation and the other with a large amount of variation. The cut point value cp. that divides these two parts was calculated to extract the conditions corresponding to Cs-rich domain. The calculation of cp. was performed using the Minimum Data Length Principle (MDLP) Discretization algorithm^44^. Specifically, we randomly selected 20 candidate values for the cut point of *I*_Cs1_, cp._*n*_ (*n* = 1, 2, …, 20), and calculated the correlation coefficient *r* between *E*_Cs1_ and *I*_Cs1_ for the data points that satisfy *I*_Cs1_ ≥ cp._n_. If *r* > 0.5, we set the label to 1, and if *r* ≤ 0.5, we set the label to 0, and then execute MDLP. The condition for the Cs-rich domain was set as *I*_Cs1_ ≥ cp. for the cp. obtained in this way. For the Sb-rich condition corresponding to the Sb-rich domain, the value of *I*_Cs1_ was set as cp._Sb_, which was the value of *I*_Cs1_ at the point where the number of data points was 1/4 of the total number of data points, from the smallest value of *I*_Cs1_ in the region where *I*_Cs1_ < cp. and *I*_Cs1_ < cp._Sb_ was set as the Sb-rich condition.

For the bootstrap method, we created a DirectLiNGAM object using the LiNGAM package in Python and used the data frame prepared by essential component extraction, calling the bootstrap method instead of the fit method. The sampling number was set to 100.

### ARPES measurements

 High-quality CsV_3_Sb_5_ single crystals were synthesized by the self-flux method. ARPES measurements were performed using a Scienta-Omicron DA30 electron analyzer at BL-28 A in Photon Factory, KEK. ARPES data shown in the main text were obtained at *T* = 8 K using circularly polarized 106-eV photons^34,35^ with a beam spot size of 10 × 12 µm^2^^45^. The energy resolution was set to be 25 meV. Additional ARPES measurements were also performed on CsV_3_Sb_5_ using an MBS-A1 electron analyzer at BL06U at NanoTerasu, and on [(PbSe)_5_][(Bi_2_Se_3_)_3_]_4_ using an MBS-A1 electron analyzer at the ANTARES beamline in SOLEIL^38^.

## Supplementary Information

Below is the link to the electronic supplementary material.

Supplementary Material 1

## Data Availability

The data supporting the findings of this study are available within the article and its Supplementary Information. All raw data are available from the corresponding author upon request.
