# Supplementary material for: Extracting causality from spectroscopy
Source: Sci Rep. 2025 Dec 22;15:43291. doi: 10.1038/s41598-025-29687-8 (PMC12722227; doi:10.1038/s41598-025-29687-8)
Supplement: Supplementary file 1 — Supplementary Material 1 [file 41598_2025_29687_MOESM1_ESM.pdf]

Supplementary information for **"Extracting causality from spectroscopy"** by Fujita *et al.*

Supplementary note 1: Number of core-level peaks

The model with more peaks (i.e., more fitting parameters) can generally provide a better fit, but also carries a risk of overfitting. Therefore, to provide an objective justification for the number of core-level peaks, we employed the Bayesian Information Criterion (BIC), which is a standard statistical method for model selection. The BIC introduces a penalty term for model complexity, thereby balancing goodness-of-fit against the number of free parameters, as detailed in literature [S1]. The BIC is defined as $2\log L + m\log N$, where L is the sum of the residual in the fitting, m is the number of parameters, and N is the number of data points. For our model consisting of K Lorentzian peaks and a linear background, the number of parameters is $m = 3K + 2$. Increasing the number of peaks (K) decreases the first term (improving the fit) but increases the second term (penalty for complexity). The most appropriate model is the one that minimizes the

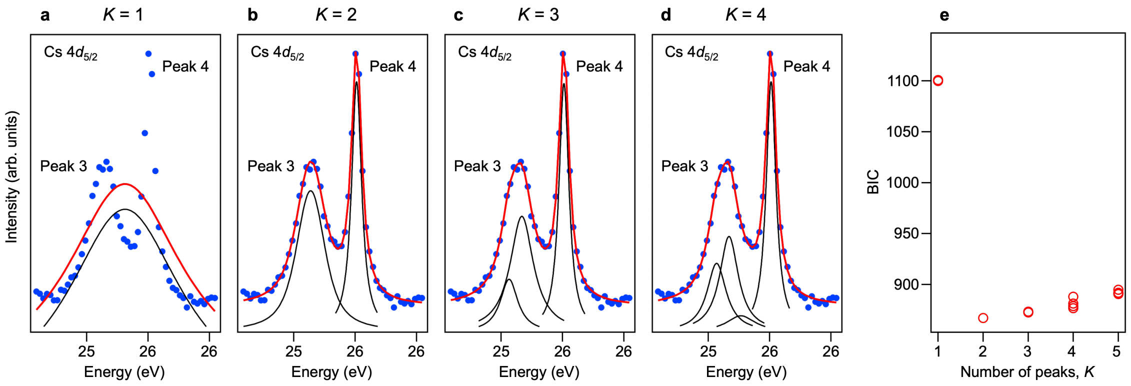

Fig. S1| Determination of number of core-level peaks. a-d Representative results of numerical fitting assuming the number of peaks K = 1, 2, 3, and 4. **e** The calculated Bayesian Information Criterion (BIC) value plotted as a function of K.

overall BIC value. Accordingly, we calculated the BIC for models with different numbers of Lorentzian peaks (*K*) combined with a linear background for the Cs 4$d_{5/2}$ satellite peaks (peaks 3 and 4) (Fig. S1a-d). As shown in Fig. S1e, the BIC value is minimized at $K = 2$ (i.e., two peaks). This result provides strong statistical support for our consideration of the two components for each spin-orbit satellite (in total four peaks for Cs 4*d* levels) and demonstrates that this number can be determined objectively.

Supplementary note 2: Correlation map for all the 14 variables in CsV_3Sb_5

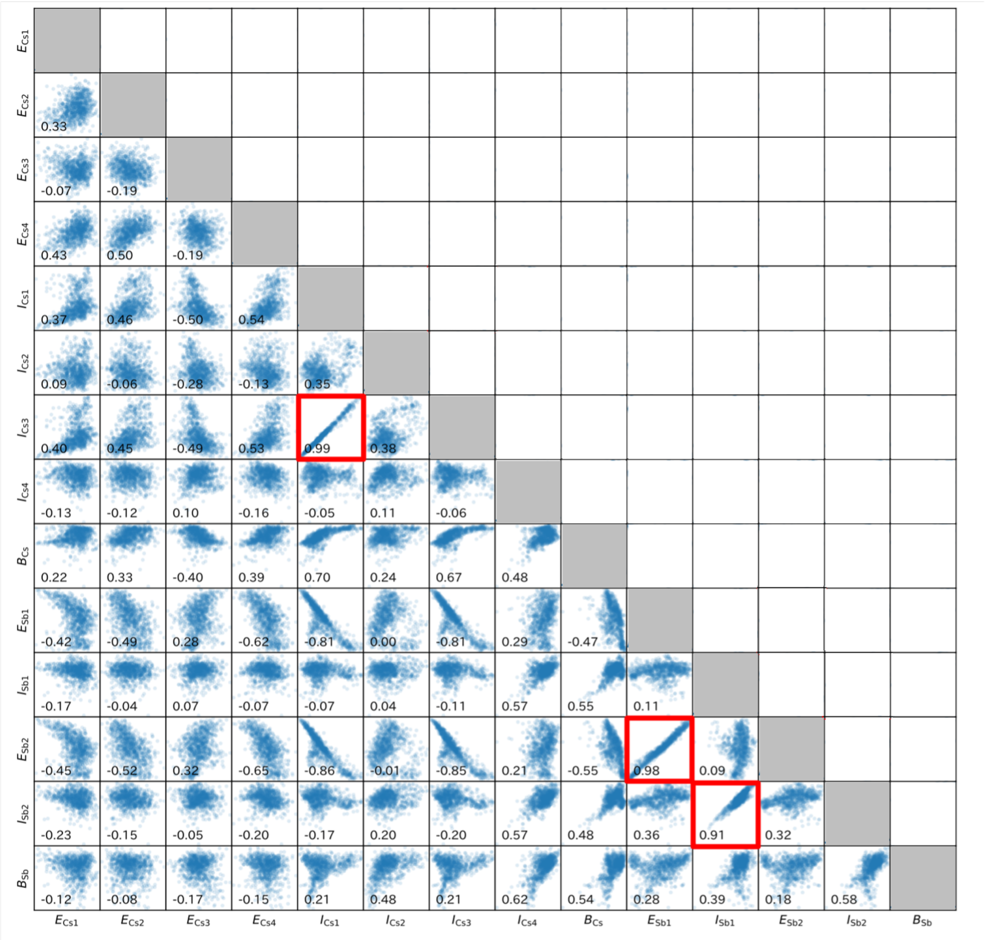

Fig. S2| Inspection of correlations among all the parameters in CsV_3Sb_5. Scatter plots of all the 14 variables extracted from the numerical fittings of Cs and Pb core-level peaks. Values in the matrix represent correlation coefficient *r*.

To visualize correlations among all the 14 parameters, we show in Fig. S2 scatter plots with 14×14 mesh for CsV_3Sb_5. While most of data points are scattered two dimensionally, one can immediately recognize a clear linear relationship in a few plots, as marked by red boxes. They correspond to the grouped pairs (I_{Cs1}, I_{Cs2}), (E_{Sb1}, E_{Sb2}), and (I_{Sb1}, I_{Sb2}), with highly correlated variables with a threshold of $r \geq 0.90$.

Supplementary note 3: Effect of arbitrary variable removal within highly correlated pairs

To evaluate whether the resulting causal graph depends on the choice of removed variable, x_i or x_j, from a highly correlated pair (x_i, x_j) with $r \geq 0.90$, we compare the causal graphs obtained after removing each variable.

Let $G(x_i)$ denote the causal graph estimated after removing x_i from the dataset. To ensure a valid comparison, the retained variables x_j in $G(x_i)$ and x_i in $G(x_j)$ were assigned as the same label "RV", so that the adjacency matrices are aligned consistently. Figure S3 shows an example of a comparison between one-variable-removed causal graphs of a highly correlated pair, I_{Cs1} and I_{Cs3}. The two graphs are similar, indicating that the causal structure remains stable regardless of the choice of removed variable.

To quantify the similarity between two one-variable-removed causal graphs, we define a similarity score f_{sim} based on the Frobenius norm of their adjacency matrices. Let A_i and A_j denote the adjacency matrices corresponding to $G(x_i)$ and $G(x_j)$, respectively, where the retained variables are aligned as described above. The similarity score is defined as

$$f_{\mathrm{sim}}(A_i, A_j) = 1 - \frac{|A_i - A_j|_F}{|A_i|_F + |A_j|_F},$$

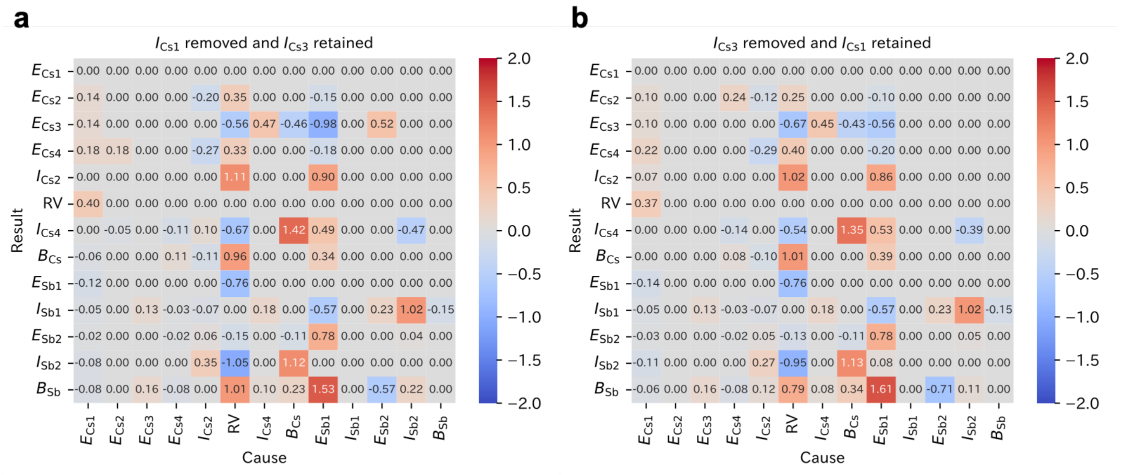

Fig. S3| Comparison of adjacency matrices corresponding to causal graphs estimated after removing either I_{Cs1} or I_{Cs3}. a,b I_{Cs1}- *and* I_{Cs3}-removed case, respectively. The retained variable is labeled as “RV” in both cases.

where the Frobenius norm is given by

$$|A|_F = \sqrt{\sum_{i,j} A_{ij}^2}.$$

The similarity score f_{sim} ranges from 0 to 1, where a value close to 1 indicates that the two causal graphs are nearly identical, whereas a value close to 0 suggests a significant difference.

To further examine the effect of variable removal across the entire dataset, we computed the similarity score f_{sim} between $G(x_i)$ and $G(x_j)$ for all possible (x_i, x_j) pairs. Figure S4a presents the obtained f_{sim} values, shown in a matrix format analogous to Fig. 3e, while Fig. S4b shows them computed from graphs whose edge weights are averaged over 1,000 bootstrap samples. The latter captures the stability of the reliability of edges in causal graphs under variable removal. As shown in Fig. S4c,d, variable pairs with high correlation tend to exhibit high similarity, indicating that removing either variable from such pairs has little effect on the causal structure.

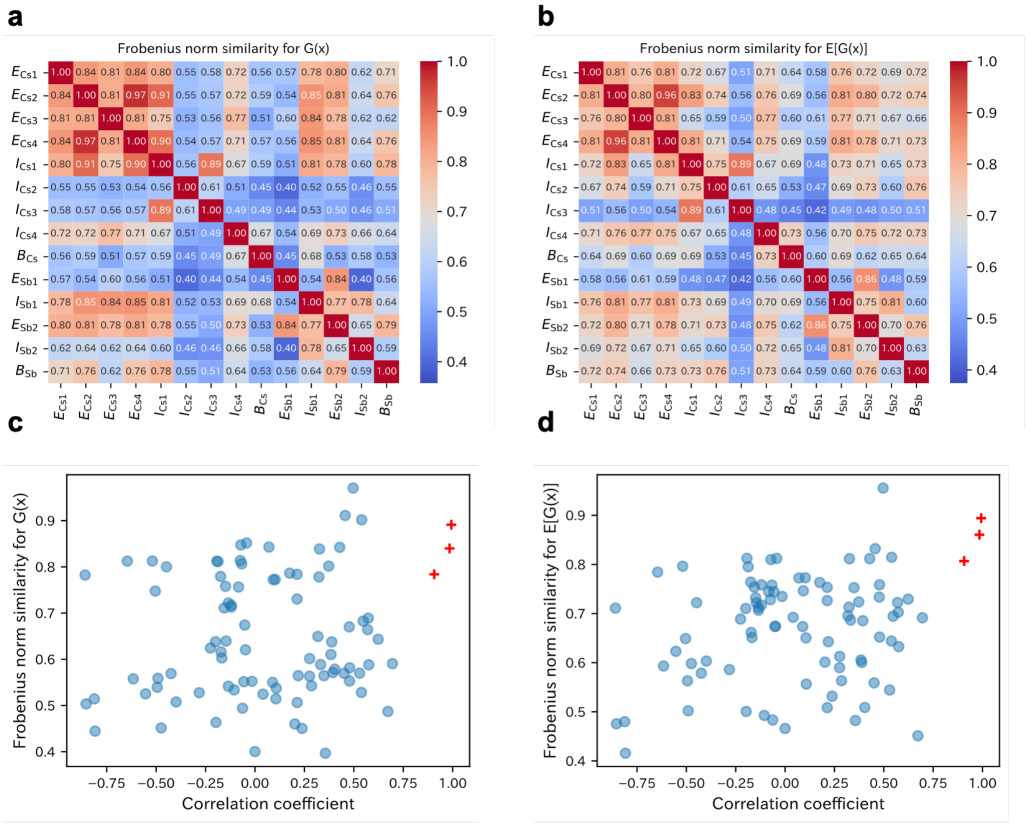

Fig. S4| Similarity between one-variable-removed causal graphs across all variable pairs. **a** Heatmap of the similarity scores f_{sim}. **b** Heatmap of f_{sim} computed from causal graphs whose edge weights are averaged over 1,000 bootstrap samples. **c**,**d** Relationship between the correlation coefficient (from Fig. 3e) and the similarity scores f_{sim} (shown in **a** and **b**, respectively). Red cross symbols denote the grouped variables satisfying $r \geq 0.9$, which consistently exhibit high f_{sim}.

Supplementary note 4: Rearrangement of node position in causal graphs

For a better comparison of common features and differences in causal graphs shown in Fig. 5, we have manually rearranged the nodes and maintained consistent positions for each variable across panels as much as possible in Fig. S5. The result clearly demonstrates that the key causal relationships—such as the edge from I_{Cs3} to E_{Sb}—are consistently present in all graphs.

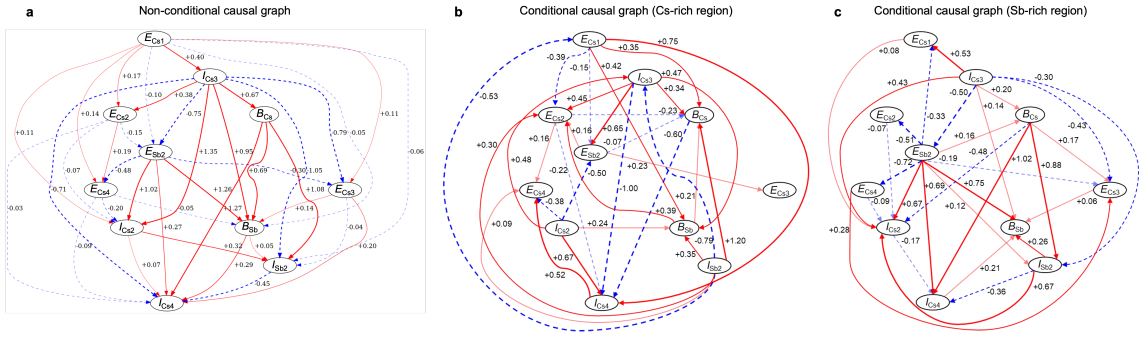

Fig. S5| Rearrangements of nodes in causal graphs. a-c Comparison of non-conditional and conditional (Cs-rich and Sb-rich regions) causal graphs. Each node in the graphs is manually placed at the same position.

Supplementary note 5: Direct and indirect causal relationships

Here we discuss indirect causal effects. In a causal graph, an indirect causal relationship from variable A to variable B is represented by paths from node A to node B that pass through one or more intermediate nodes. The indirect causal effect along a specific path is calculated by multiplying the weights of all edges comprising that path. Figure S6a-c shows adjacency matrices representing direct causal effects in the full, Cs-rich, and Sb-rich regions, respectively (Fig. S6a is identical to Fig. 4a in the main text), while Fig. S6d-f shows corresponding total causal effect matrices including both direct and indirect effects, whose entries represent sums of causal effects for all paths between the cause node and the result node. The similarity of these matrices indicates that indirect causal effects are minor in most pair-wise causal relationships. As mentioned in the main text, the direct causality from I_{Cs3} to E_{Sb2} is generally large in all three domains (full, Cs-rich, and Sb-rich). This is also the case when indirect effects are taken into account.

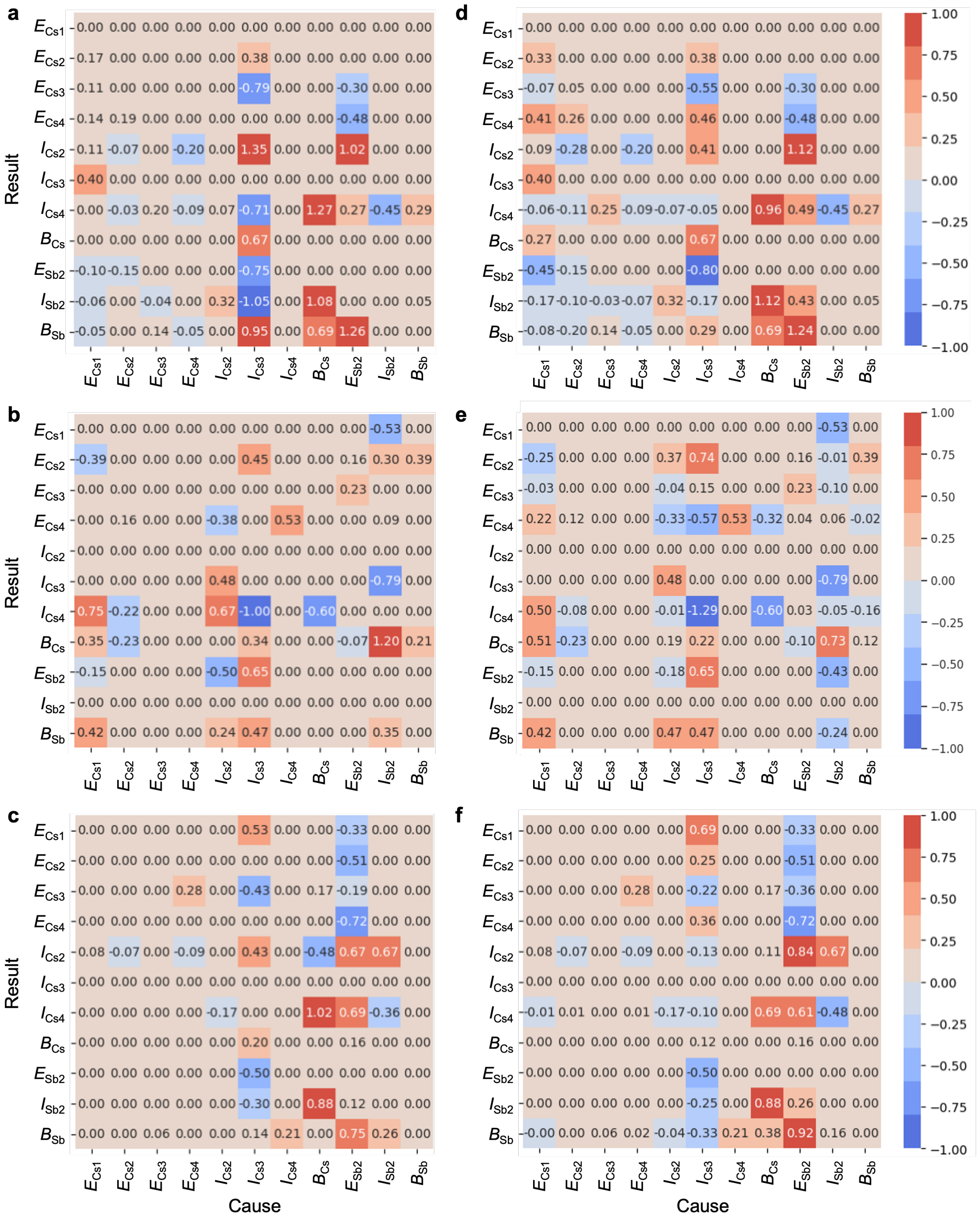

Fig. S6| Comparison of adjacency matrices and causal effect matrices including direct and indirect effects in full region, Cs-rich, and Sb-rich, respectively. a-c Adjacency matrices of full, Cs-rich, and Sb-rich regions, respectively, obtained by assuming only direct causality. **d-f** Same as **a-c**, respectively, but obtained by considering both direct and indirect effects.

Supplementary note 6: Influence of fitting/analysis errors on causality

To test the robustness of our analysis against measurement and analysis errors, we created a new dataset where the noise level of photoemission spectra was intentionally increased. Specifically, we used only half of the original photoemission data for spectral integration at each spatial location, thereby effectively lowering the signal-to-noise ratio. We then performed the entire causal discovery analysis on this new dataset. The result, shown in Fig. S7, demonstrates that the primary causal relationships identified in our original analysis (Fig. 5a), such as the causal link from I_{Cs3} to E_{Sb2}, are successfully reproduced. These findings indicate that the variation present in variables is sufficiently small to allow an accurate estimation of reliable causal relationships.

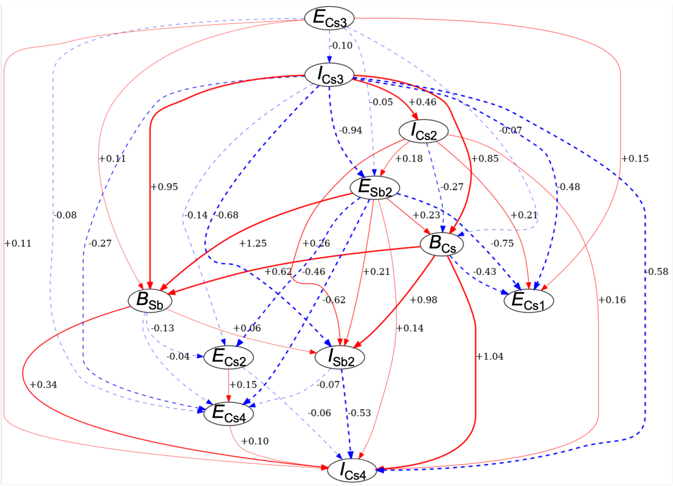

Fig. S7| Influence of fitting/analysis errors on causality. Causal graph extracted from the analysis using only half of the data.

Supplementary note 7: Causal discovery using other algorithms

We performed additional causal analyses using the Peter-Clark (PC) algorithm [S2] and Greedy Equivalence Search (GES) [S3]. The results are shown in Fig. S8. Our comparison indicates that LiNGAM is better suited for analyzing the present system. For

example, the well-established causal link from I_{Cs3} to E_{Sb2} is correctly identified by LiNGAM. In contrast, both PC and GES algorithms fail to capture this known physical relationship correctly; in their output graphs, I_{Cs3} appears downstream of E_{Sb2} as an effect. In addition, LiNGAM provides a fully directed graph, which makes it more powerful for generating specific and testable causal hypotheses.

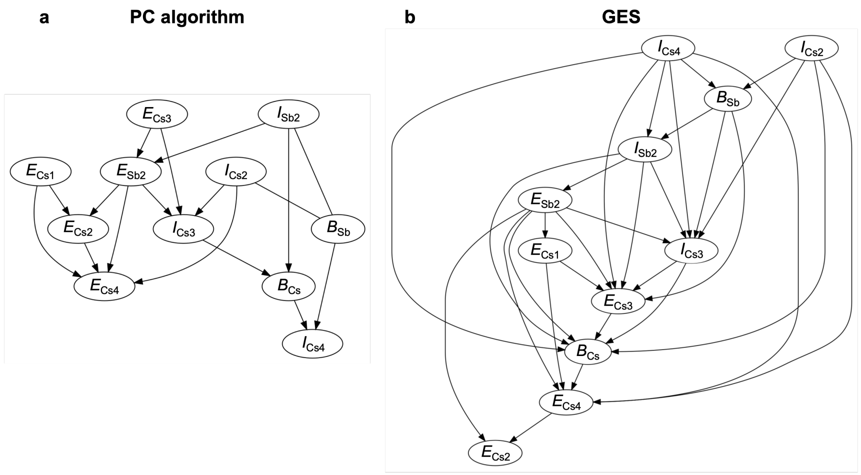

Fig. S8| Causal discovery using different algorithms. a,**b** Comparison of causal graphs obtained by the Peter-Clark (PC) and Greedy Equivalence Search (GES) algorithms.

Supplementary note 8: Causal inference with a different fitting model

To rule out the possibility of a fitting flaw as the origin of the unexpected relationship between I_{Cs2} and I_{Cs4}, we conducted additional analysis that goes beyond the relatively simple model of Lorentzian peaks with a linear background used in the main text. Specifically, we used a more sophisticated fitting model using Doniach-Sunjic functions [S4], which capture the intrinsic asymmetry of core-level peaks in metals, combined with a Shirley-type background [S5] that incorporates the shape of the density of states. As shown in Fig. S9a, this model reproduces the experimental spectra very well. Importantly, despite the improved fit, I_{Cs2} and I_{Cs4} exhibit a low correlation [−0.01 in the correlation

matrix in Fig. S9b; note that the correlation of their energy positions, E_{Cs2} and E_{Cs4}, remains moderate (~0.5)]. This strongly suggests that the anomalous intensity ratio is an intrinsic feature of the data and not an artifact of an inadequate fitting procedure.

This is further supported by the fact that constraining the ratio of I_{Cs2} to I_{Cs4} to 2:3 clearly fails to reproduce the experimental result in the Sb-rich domain (Fig. S9c), while it shows better agreement with the data in the Cs-rich domain (Fig. S9d). This indicates that the peak weight ratio is not constant (also see the scatter plot I_{Cs2} vs I_{Cs4} in Fig. S9e, where data points largely spread out over a wide range of weight ratio).

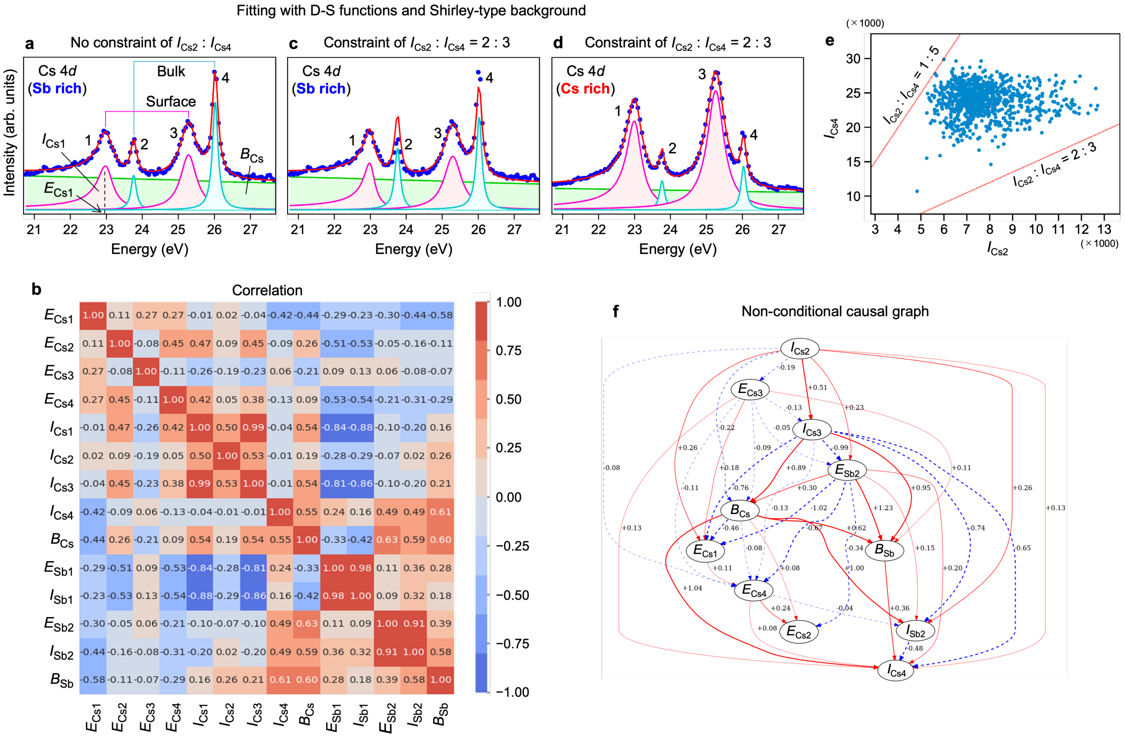

Fig. S9| Causal inference with a different fitting model. **a** Cs 4*d* core-level spectrum (dots), with the result of numerical fittings (solid curves) with four Doniach-Sunjic (D-S) functions and Shirley-type background. **b** Correlation matrix obtained with the fitting results using D-S functions and Shirley-type background. **c** The result of numerical fittings in the Sb-rich domain assuming I_{Cs2}:I_{Cs4} = 2:3. **d** Same as **a**, but in the Cs-rich domain. **e** Causal graph extracted from the fitting results using D-S functions and a Shirley-type background.

We also regenerated the causal graph using the variables extracted from the fitting model used here (Fig. S9f). We found that the main causal relationships discussed in the main text (Fig. 5a), such as the causal link from I_{Cs3} to E_{Sb2}, are well reproduced. These findings confirm that the causal graph reliably extracts the essential information, remaining robust against the choice of fitting model.

Supplementary note 9: Causal discovery in a topological superlattice

To clarify the broader applicability of our causal discovery scheme, we have examined causal relationships in a topological superlattice $[(PbSe)_5][(Bi_2Se_3)_3]_4$ (called here PSBS) where previous nano-ARPES measurements revealed clear domain-dependent core-level intensities [S6]. PSBS consists of an alternate stacking of four quintuple layers (4QLs) of Bi_2Se_3–a topological insulator building block-and one bilayer (1BL) of PbSe with rock-sault structure [S6,S7]. Upon cleavage, either 1QL or 2QL Bi_2Se_3 remains on top of the PbSe layer (3QL domains are rare [S7]) as shown in Fig. S10a. As shown in Fig. S10b, the 1QL and 2QL domains on the cleaved surface are readily distinguished by the intensity ratio of Bi 5*d* spin-orbit satellite peaks (B1 and B2 in the inset to Fig. S10b) to Pb 5*d* peaks (Pb1 and Pb2). This contrast originates from the reduced photoelectron emission from the buried PbSe layer in 2QL domains due to the short mean-free path of photoelectrons.

In Step 1, we fitted the Bi1, Bi2, Pb1, and Pb2 peaks with four Lorentzian peaks and a linear background, extracting their peak positions (E_{Bi1}, E_{Bi2}, E_{Pb1}, E_{Pb2}), spectral weight (I_{Bi1}, I_{Bi2}, I_{Pb1}, I_{Pb2}), and background intensity (B).

In Step 2, we calculated correlation coefficients (r) between all 9 input variables (Fig. S10c). One can identify strongly correlated pairs: (E_{Bi1}, E_{Bi2}) with $r = 1.0$, (I_{Bi1}, I_{Bi2}) with

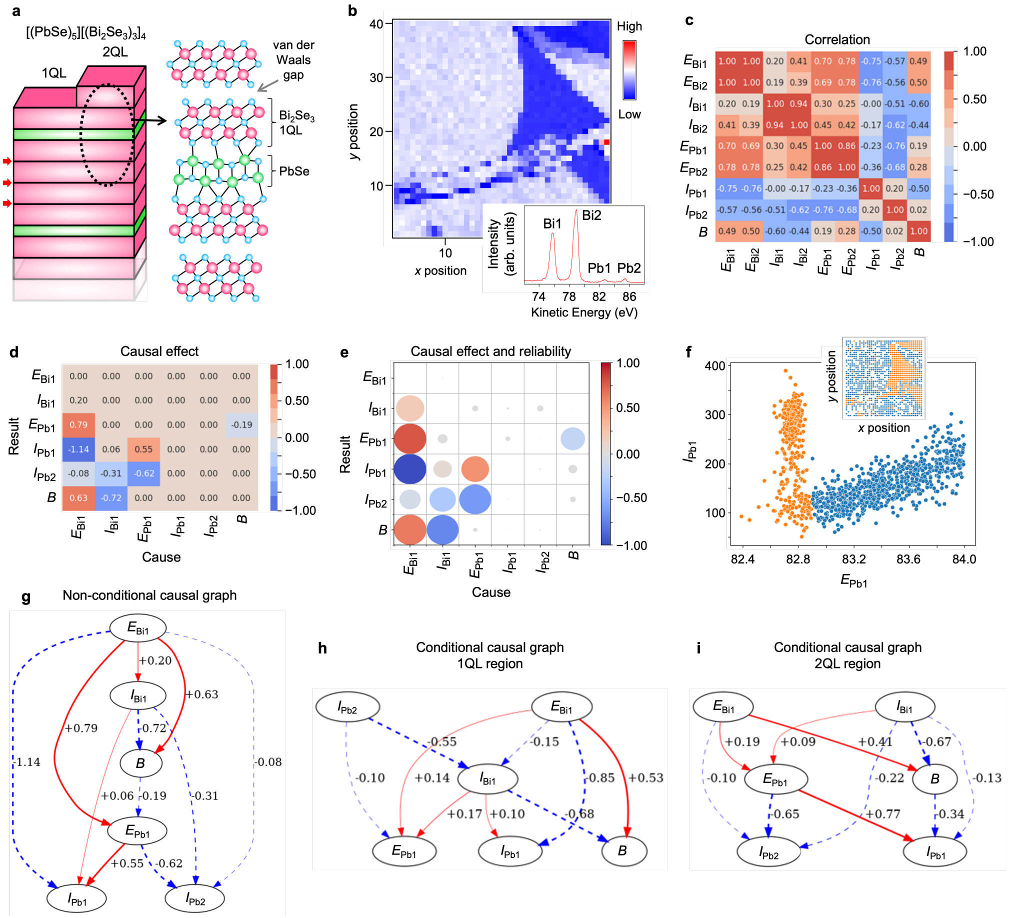

Fig. S10| Extracting causality in a topological superlattice. a, Side view of the crystal structure of $[(PbSe)_5][(Bi_2Se_3)_3]_4$ (PSBS) [S7], showing alternate stacks of four Bi_2Se_3 quintuple layers (QLs) and one PbSe bilayer (BL). Red arrows indicate cleavage planes. **b**, Spatial map of total spectral weight of Bi 5*d* core levels with respect to that of Pb 5*d* core levels. Inset shows the energy distribution curve in the kinetic energy range of 72-88 eV covering Bi 5*d* and Pb 5*d* core levels. **c**, Correlation matrix of input variables: Bi core-level peak energies (E_{Bi1} and E_{Bi2}) and spectral weights (I_{Bi1} and I_{Bi2}); Pb core-level energies (E_{Pb1} and E_{Pb2}) and spectral weights (I_{Pb1} and I_{Pb2}); and background intensity (B). **d**, Adjacency matrix of causal effects between “result” and “cause” variables, after removing E_{Bi2}, I_{Bi2}, and E_{Pb2}. **e**, Same as **d**, but the magnitude of causal effects is highlighted by the gradual color shading of circles. Circle sizes indicate causal relationship probability in bootstrap resampling. **f**, Scatter plot of (E_{Pb1}, I_{Pb1}) pair, which was used to distinguish 1QL (orange dots) and 2 QL (blue dots) domains. Inset shows the spatial map of 1QL and 2QL domains, which signifies a good agreement with the intensity map in **b**. **g-i**, Causal graphs for the full (x, y) region, 1QL domain, and 2QL domain, respectively.

$r = 0.94$, and (E_{Pb1}, E_{Pb2}) with $r = 0.86$. These strong correlations are consistent with the spin-orbit partner nature of each pair [in this regard, low r value (0.11) for (I_{Pb1}, I_{Pb2}) was unexpected]. We have grouped these highly correlated variables and removed E_{Bi2}, I_{Bi2}, and E_{Pb2} to reduce variables.

In Step 3, we analyzed causal relationships from the 6 variables and generated the adjacency matrix (b values) (Fig. S10d) and corresponding color-coded circle representation in which the circle radii indicate the probability of causal relationships (Fig. S10e). When the absolute b value is high, the probability is also high, supporting the validity of the data sampling as in the case of CsV_3Sb_5 (Fig. 4). E_{Bi1} is a primary key parameter of a "cause" variable, and I_{Bi1} or E_{Pb1} emerges as secondary "causes". Notably, 25 out of 36 (6×6) b values (69%) are zero. I_{Pb1} and I_{Pb2} exhibit no causal influence as "causes".

To draw conditional causal graphs, we distinguished 1QL and 2QL domains based on the E_{Pb1} vs I_{Pb1} scatter plot (Fig. S10f), in which two groups (orange and blue circles) can be categorized by E_{Pb1} variations as expected from the depth of PbSe layer and the short photoelectron mean-free path. The similar triangular 1QL domain shape in Fig. S10b and the inset of Fig. S10f supports successful domain categorization.

Common features are observed across non-conditional and conditional causal graphs (Fig. S10g-i). Notably, E_{Bi1} is situated at the top of the graphs and emits multiple edges, indicating its high causal order. Specifically, an edge (red arrow; i.e. positive causal effect) from E_{Bi1} to E_{Pb1} is present in all graphs, reflecting that larger Bi core-level energy shits (thicker Bi_2Se_3 layer) enhance the Pb core-level energy shifts, consistent with the successful domain categorization based on E_{Pb1}. Similarly, an edge from E_{Bi1} to B is observed in all graphs, suggesting that local chemical bonding and carrier doping, as

reflected by surface core-level shifts, significantly affect the background intensity. This is somewhat unexpected from a simple secondary electron background perspective. These findings suggest the applicability of our causal discovery scheme to topological materials, demonstrating its versatility in a variety of material systems.

References

S1. Shinotsuka, H. Full automatic peak separation of X-ray photoelectron spectroscopy spectra. *J. Electron Spectrosc. Relat. Phenom.* **239**, 146903 (2020).

S2. Spirtes, P., Glymour, C. N., Scheines, R. & Heckerman, D. Causation, prediction, and search. *MIT press*. (2000).

S3. Chickering, D. M. Optimal structure identification with greedy search. *J. Mach. Learn. Res.* **3**, 507 (2002).

S4. Doniach, S. & Sunjic, M. Many-electron singularity in X-ray photoemission and X-ray line spectra from metals. *J. Phys. C: Solid State Phys.* **3**, 285 (1970).

S5. Shirley, D. A. High-Resolution X-Ray Photoemission Spectrum of the Valence Bands of Gold. *Phys. Rev. B* **5**, 4709 (1972).

S6. Nakayama, K., Eto, K., Tanaka, Y., Sato, T., Souma, S., Takahashi, T., Segawa, K. & Ando, Y. Manipulation of Topological States and the Bulk Band Gap Using Natural Heterostructures of a Topological Insulator. *Phys. Rev. Lett.* **109**, 236804 (2012).

S7. Nakayama, K., Souma, S., Trang, C. X., Takane, D., Chen, C., Avila, J., Takahashi, T., Sasaki, S., Segawa, K., Asensio, M. C., Ando, Y. & Sato, T. Nanomosaic of Topological Dirac States on the Surface of $Pb_5Bi_{24}Se_{41}$ Observed by Nano-ARPES. *Nano Lett.* **19**, 3737 (2019).
